# Revealing inscriptions obscured by time on an early-modern lead funerary cross using terahertz multispectral imaging

**DOI:** 10.1038/s41598-022-06982-2

**Published:** 2022-03-02

**Authors:** Junliang Dong, Ana Ribeiro, Aurélien Vacheret, Alexandre Locquet, D. S. Citrin

**Affiliations:** 1 Georgia Tech-CNRS IRL 2958, Georgia Tech Lorraine, 2 Rue Marconi, Metz, 57070 France; 2 School of Electrical and Computer Engineering, Georgia Institute of Technology, Atlanta, Georgia, 30332-0250 USA; 3 Laboratoire d’Archéologie des Métaux, 1 Avenue du Général de Gaulle, 54140 Jarville-la-Malgrange, France; 4 Musée Charles de Bruyères, 70 rue Charles de Gaulle, 88200 Remiremont, France; 5 Present Address: Institut national de la recherche scientifique, Centre Énergie Matériaux Télécommunications, Varennes, QC J3X 1P7 Canada

**Keywords:** Applied optics, Optical techniques

## Abstract

The presence of a corrosion layer on lead art and archæological objects can severely impede the interpretation of inscriptions, thus hampering our overall understanding of the object and its context. While the oxidation of lead that dominates corrosion may be chemically reversible via reduction, potentially providing some access to inscriptions otherwise obscured by time, corrosion damage is overall neither entirely reversible nor is the reduction process in all cases easy or feasible to carry out. In this study, by taking advantage of the unique penetration ability of terahertz radiation and the abundant frequency bands covered by a single-cycle terahertz pulse, we perform nondestructive terahertz multispectral imaging to look under the corrosion on a sixteenth century lead funerary cross (*croix d’absolution*) from Remiremont in Lorraine, France. The multispectral images obtained from various terahertz frequency bands are fed into a judiciously designed post-processing chain for image restoration and enhancement, thus allowing us for the first time to read obscured inscriptions that might have otherwise been lost. Our approach, which brings together in a new way the THz properties of the constituent materials and advanced signal- and image-processing techniques, opens up new perspectives for multi-resolution analysis at terahertz frequencies as a technique in archæometry and will ultimately provide unprecedented information for digital acquisition and documentation, character extraction, classification, and recognition in archæological studies.

## Introduction

Since prehistoric times, objects have been included in burials to facilitate the passage to the next world. Such practices persisted through the Middle Ages in Europe where a number of types of grave goods have been found specifically addressing the Christian’s passage (hopefully) to heaven. In particular, cross-shaped plaques, cut from thin lead (Pb) sheets have been found at a number of sites in France, Germany, and England^1^, with a concentration in Lorraine, France (now within the Région du Grand Est) where the practice continued to modern times (eighteenth century). These Pb crosses were found on the deceased’s chest within the coffin. The crosses themselves typically contain a prayer and/or information about the deceased incised into the cross, and the putative purpose was to seek absolution from sin. A particularly large find^2^ was in Remiremont in Département des Vosges, and this study involves a Pb funerary cross dating from the sixteenth century (cross No. 28 as reported in Ref.^2^). An abbey was founded in Remiremont in the seventh century and extensive burials connected with the abbey were excavated in 1843 at which time the Pb cross studied here was found.

**Figure 1:**
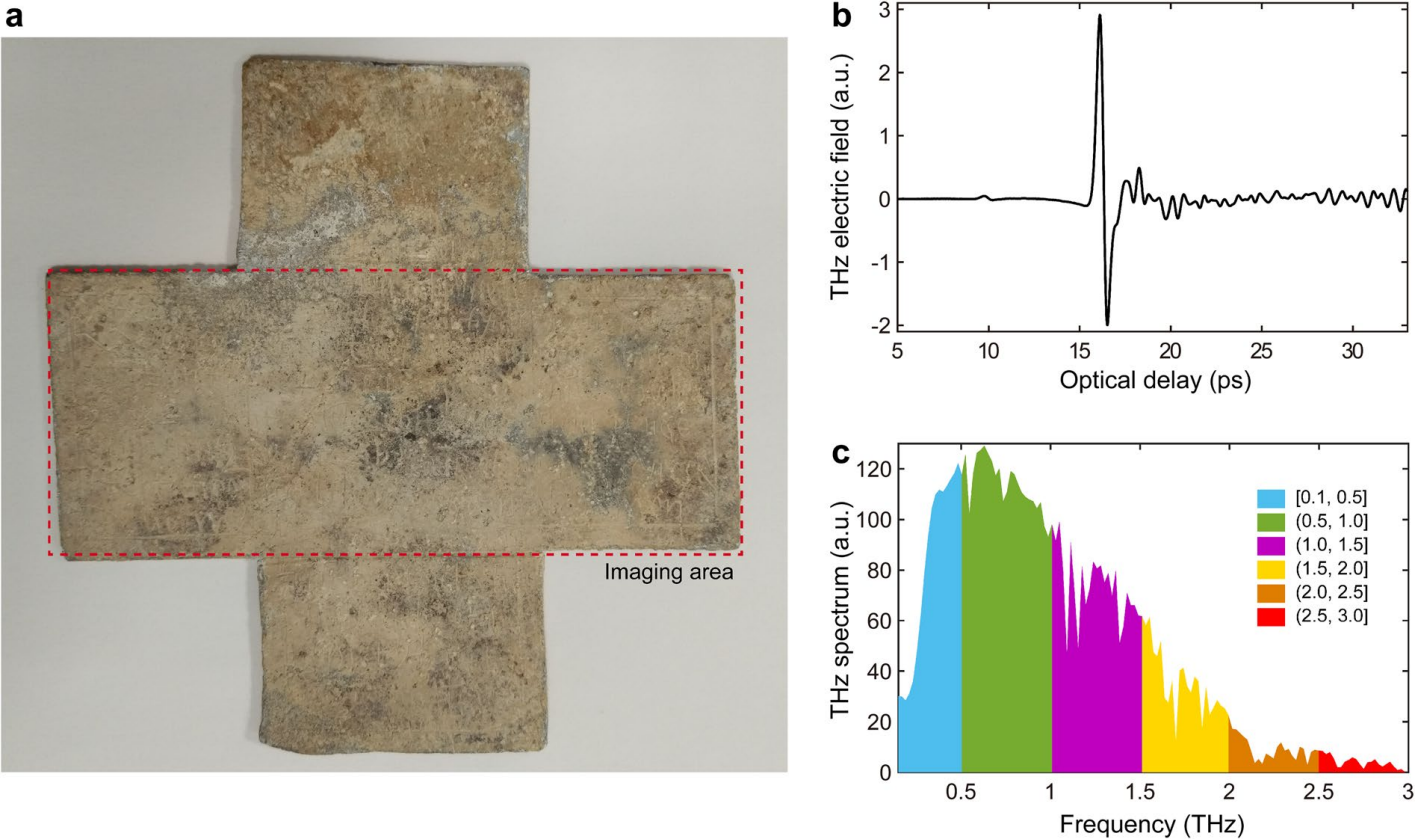
(**a**) Photograph of the Pb cross under investigation in this study. The dimensions of the cross are $\sim$11.5 cm by $\sim$11.5 cm with a thickness of $\sim$2 mm. The whitish appearance is due to corrosion. The presence of a text is suggested by incised features in the whitish areas with several characters readable where the corrosion is less well-formed. This is the condition in which the cross arrived in our laboratory. The horizontal arm of the cross is selected as the region of interest for THz imaging. (**b**) The THz reference signal in our system, which is recorded by setting a metal plate at the sample position. (**c**) The power spectrum of the reference THz pulse. The full spectrum of a broadband THz pulse is divided into six frequency bands with a bandwidth of $\sim$0.5 THz. THz multispectral images are obtained based on the frequency components in each frequency band.

The Pb cross investigated in this study is shown in Fig. 1a. The dimensions are $\sim$11.5 cm by $\sim$11.5 cm and $\sim$2 mm thick. The whitish appearance of the cross is due to the formation of corrosion products of Pb, which are probably lead carbonates^3^ [such as cerussite $\hbox {PbCO}_3$, hydrocerussite $\hbox {Pb}_3$($\hbox {CO}_3$)$_2$(OH)$_2$ or plumbonacrite $\hbox {Pb}_5$O(OH)$_2$($\hbox {CO}_3$)$_3$] or lead oxides^3,4^ [such as massicot $\beta$-PbO ([orthorhombic lead (II) oxide])]. Some reddish areas may be due to the presence of a second phase, $\alpha$-PbO [trigonal lead (II) oxide (litharge)]. The presence of a text is suggested by incised features in the whitish areas with a few characters barely readable in the greyer region where the corrosion layer is not as well-formed. When documented in 1904, Chevreux notes that only a small part of the text could be read and he remarks about this cross, “*Très peu lisible*”^2^, which means in effect “very hard to read”. In the corroded state under which the cross arrived in our laboratory, the inscription was indeed almost unreadable. While the lead carbonates and oxides can be reduced in CO to metallic Pb, the overall damage due to corrosion is not itself reversible. Also, in fragile objects and/or objects that must remain in situ, this process may not be feasible. Noninvasive and noncontact imaging modalities operating at various frequencies, such as infrared photography^5^, multispectral (between 550 nm and 950 nm) imaging^6^, as well as X-ray fluorescence imaging^7^, are under active research to improve the legibility of ancient inscriptions. However, such modalities are not suitable for the investigation of corroded Pb objects, because conventional optical frequencies cannot penetrate the corrosion layer; X-rays are strongly absorbed by Pb and may not be able to distinguish the corrosion products from the underlying Pb.

Recently, imaging with terahertz (THz) radiation^8^ has attracted considerable attention due to its unique ability to penetrate many electrically insulating materials that are opaque to conventional optical frequencies. The THz frequency range spans from 100 GHz to 10 THz, straddling the boundary between microwaves and infrared light. In particular, unlike X-rays, THz radiation is nonionizing and thus presents no health risks^9^. Until now, THz time-domain imaging^10^ has been explored to characterize the structure and chemical composition of objects of archæological^11^ and art-historical^12^ interest, such as paintings^13,14^, ceramics^15^, stones^16^, bronzes^17^, seals^18^, manuscripts^19,20^, as well as mummies^21,22^. In this study, we investigate the potential of THz pulsed imaging in reflection mode to reveal the obscured inscriptions under corrosion on the sixteenth century Pb funerary cross. By exploiting the broadband nature of a single-cycle THz pulse, we are able to acquire THz multispectral images^23–26^ from different frequency bands, in turn allowing us to characterize the surface and subsurface features of the Pb cross with different spatial resolutions. In particular, our results demonstrate that the features associated with the inscriptions can only be identified in the high-frequency band, and the additional information provided by the low-frequency band can be input into our judiciously designed image post-processing chain to enhance the visibility of the inscriptions. Our strategy can be applied to a wide range of cultural heritage objects and provide invaluable information for archæological studies, as well as potentially for documentation, conservation, and restoration. Our work thus brings together in a novel combination the science of THz-materials interactions with signal and image processing, to bear on a problem in archæological science.

## Results

### Experiment

The Pb cross sample shown in Fig. 1a was investigated via THz pulsed imaging based on a typical THz time-domain spectroscopy system (see “Methods”). THz pulsed imaging involves launching roughly single-cycle THz pulses to access both the surface and subsurface information of the Pb cross, here applied in a reflection geometry. The variations in THz reflections, corresponding to the irregularities/discontinuities/roughness of the air/corrosion and corrosion/Pb interfaces, contains the information we seek related to the inscription. Figure 1b displays the reference THz pulse of our system, recorded by setting a metal plate at the sample position. The THz pulse exhibits a power spectrum extending from $\sim$60 GHz to 3.0 THz, as shown in Fig. 1c. The THz spot size is frequency-dependent^27^ and its full-width at half-maximum (FWHM) intensity is $\sim$2.42 mm at 1 THz. We selected the horizontal arm of the Pb cross as the region of interest, shown in Fig. 1a, which was raster-scanned with a set of motorized stages moving in the X and Y directions with a 0.2-mm step. After the raster-scans, a 3D volume of reflected data was acquired.

**Figure 2:**
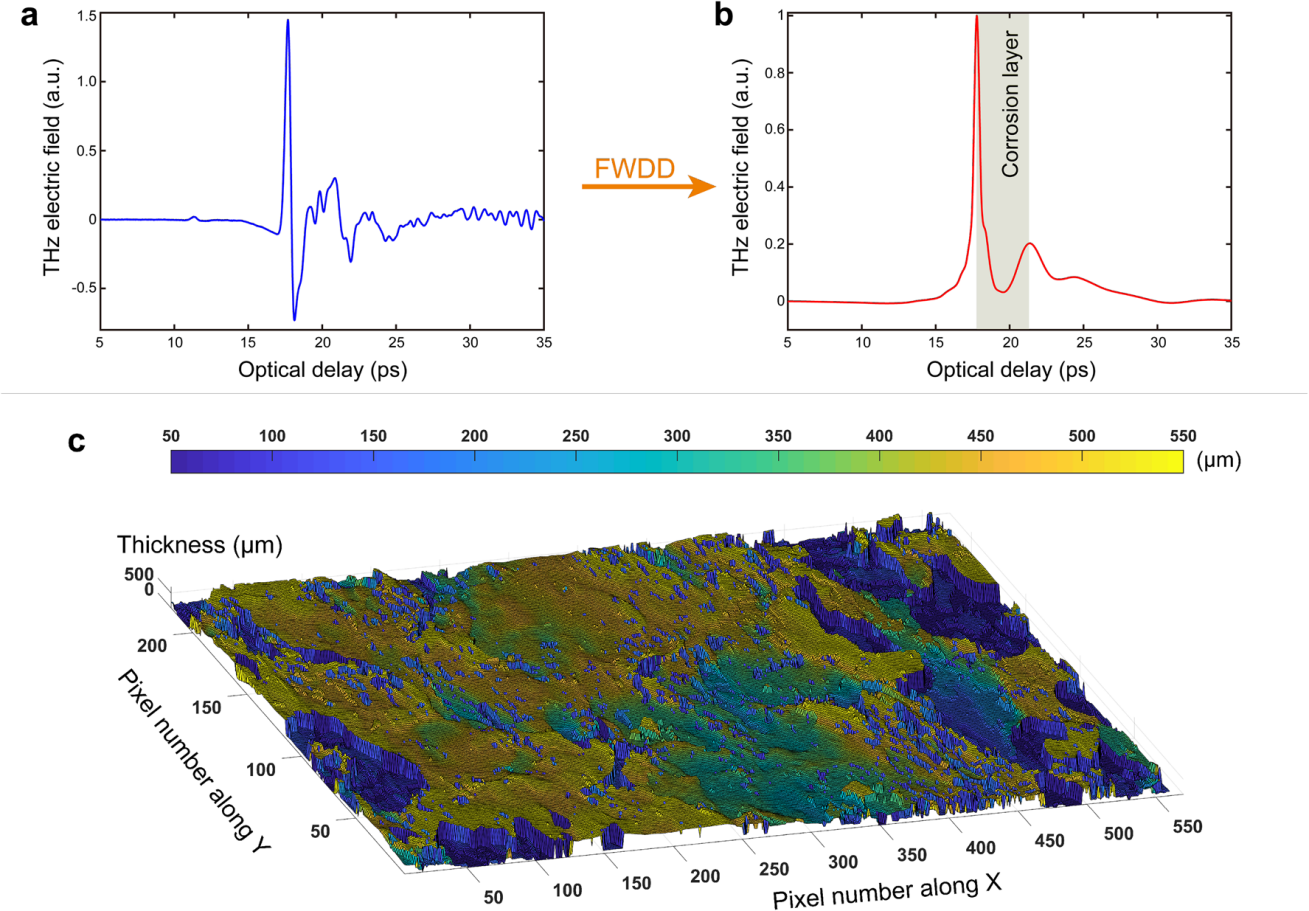
(**a**) The THz reflected signal acquired at pixel (250, 190). The THz reflected signal mainly contains two echoes corresponding to the reflections from the air/corrosion and corrosion/Pb interfaces. (**b**) Deconvolved signal after performing the FWDD algorithm. The two positive peaks indicate the location of the interface, and the optical delay between the two peaks corresponds to the corrosion thickness. (**c**) The distribution of the corrosion thickness across the Pb cross.

### Corrosion thickness mapping

In principle, the incident THz pulses are partially reflected at the air/corrosion interface, but a portion of the power will transmit into the corrosion layer to be reflected from the corrosion/Pb interface. Therefore, the received THz signals are the superposition of the two echoes reflected from the air/corrosion and corrosion/Pb interfaces with a relative time delay^28^. A typical THz reflected signal at pixel (250, 190) is shown in Fig. 2a. We use the frequency-wavelet domain deconvolution (FWDD) algorithm (see “Methods”) to process the THz reflected signals in order to accurately measure the time delay between the two echoes. As shown in Fig. 2b, after performing the FWDD, two positive peaks are clearly identified, corresponding to the locations of the air/corrosion and corrosion/Pb interfaces. The optical thickness of the corrosion at such a given position can be acquired by measuring the optical delay between the two peaks, and the physical thickness of the corrosion can be further calculated upon the knowledge of the refractive index of the corrosion in the THz regime.

As visually evident in Fig. 1a, the corrosion layer is inhomogeneous, with a spatially varying composition and thickness. The refractive indices of various lead carbonates and oxides at THz frequencies have not yet been reported. A value for the refractive index of ground massicot pigment in an animal-glue binder is $\sim$1.50^29^. This is an effective value for the dried paint, and the actual value for massicot is likely substantially larger. At optical frequencies, the value for the refractive index is  2.55^30^. In view of the uncertainty, we assume a representative value of 2.5 for the estimation of the corrosion thickness; the results shall show that this assumption, if not ideal, provides a working value leading to successful reading of the sub-corrosion inscription. After processing the 3D volume THz reflected data, the distribution of corrosion thickness across the Pb cross is estimated and displayed in Fig. 2c. There are some areas in which the thickness is not successfully acquired, because the signal-to-noise ratio (SNR) there is quite low, in turn hampering our effort to detect a valid second echo. We note that no features associated with the inscriptions can be identified in the map of corrosion thickness. This is because the time-domain information cannot provide enough spatial resolution to reveal subtle features.

### THz multispectral images

**Figure 3:**
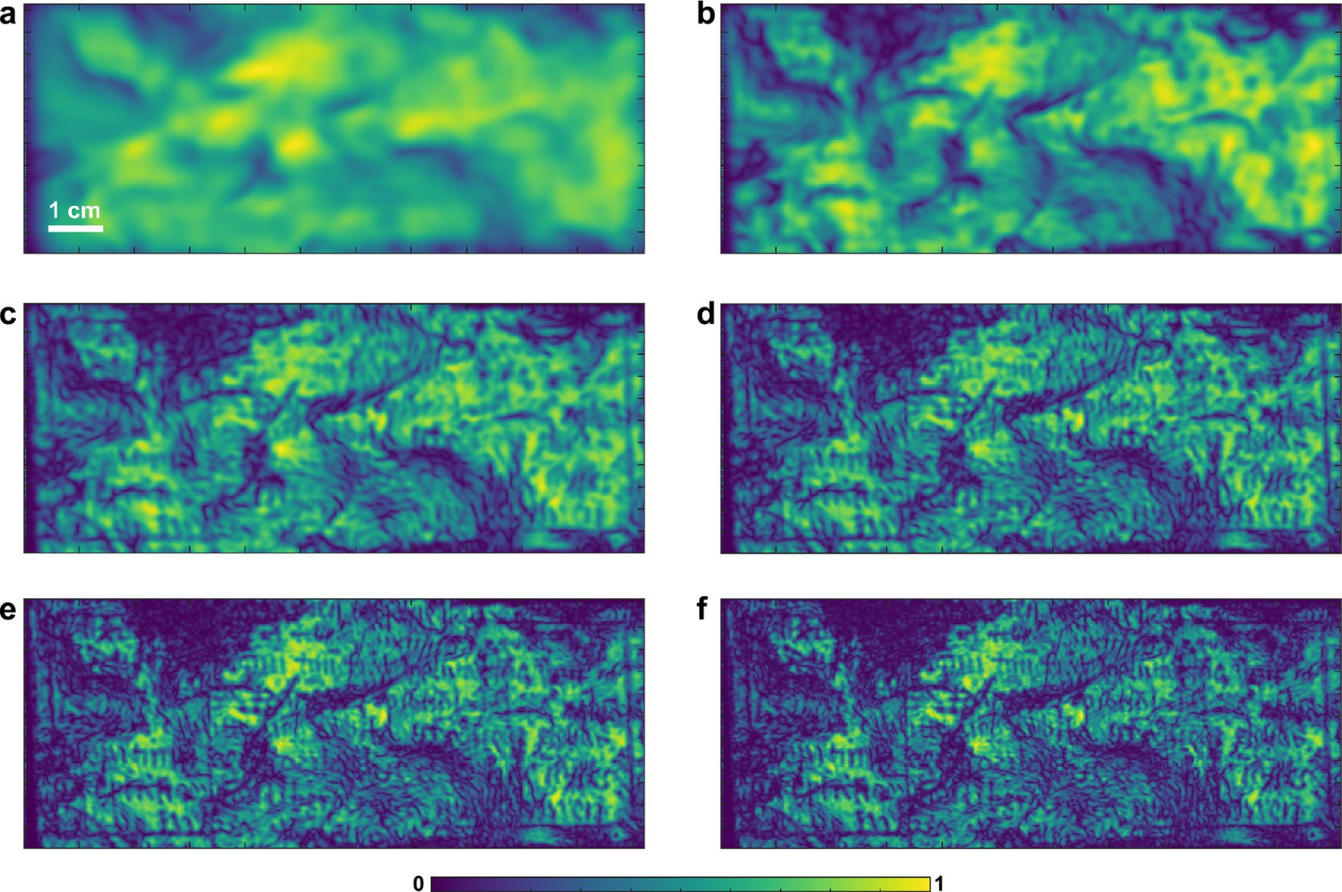
THz multispectral images obtained based on the components in frequency bands within (**a**) 0.1 and 0.5 THz, (**b**) 0.5 THz and 1.0 THz, (**c**) 1.0 THz and 1.5 THz, (**d**) 1.5 THz and 2.0 THz, (**e**) 2.0 THz and 2.5 THz, (**f**) 2.5 THz and 3.0 THz. Features associated with the inscriptions start to appear in the frequency bands above 1 THz, and images from the low frequency bands mainly reflect the surface morphology of the cross. All the images are normalized to their maximum pixel values.

Based on the broadband nature of a single-cycle THz pulse, we are able to characterize the Pb cross with different spatial resolutions using different THz frequency components. THz multispectral images can be obtained by taking the Fourier transform of the reflected THz signal *E*(*t*) at each pixel, and integrating the magnitude of the frequency components within various bands. The imaging contrast mechanism *C* at a specific pixel is defined as

1 $$\begin{aligned} C = \int _{f_l}^{f_h} \left| FFT(E(t))\right| df, \end{aligned}$$

where $f_l$ and $f_h$ are the limits of the exploited frequency band, and *FFT* represents the fast Fourier transform. In our study, we divide the full bandwidth provided by the THz pulse into six frequency bands, each of which has a bandwidth of $\sim$0.5 THz, as shown in Fig. 1c. Based on Eq. (1), THz multispectral images achieved from six frequency bands are plotted in Fig. 3. It is clear that THz multispectral images are effective in revealing the surface roughness, as well as evidence of subsurface features. In the presence of irregularities at the interface, enhanced scattering of THz signals ensues, and consequently leading to a weaker specular signal. As shown in Fig. 3, markings corresponding to the incised inscriptions start to appear in the frequency band above 1 THz and become more obvious as the frequency increases, whereas images in the lower frequency bands mainly reveal the surface morphology of the cross. This is because higher frequency components, corresponding to shorter wavelengths, bring out small and subtle features associated with the inscriptions.

### Image post-processing chain

**Figure 4:**
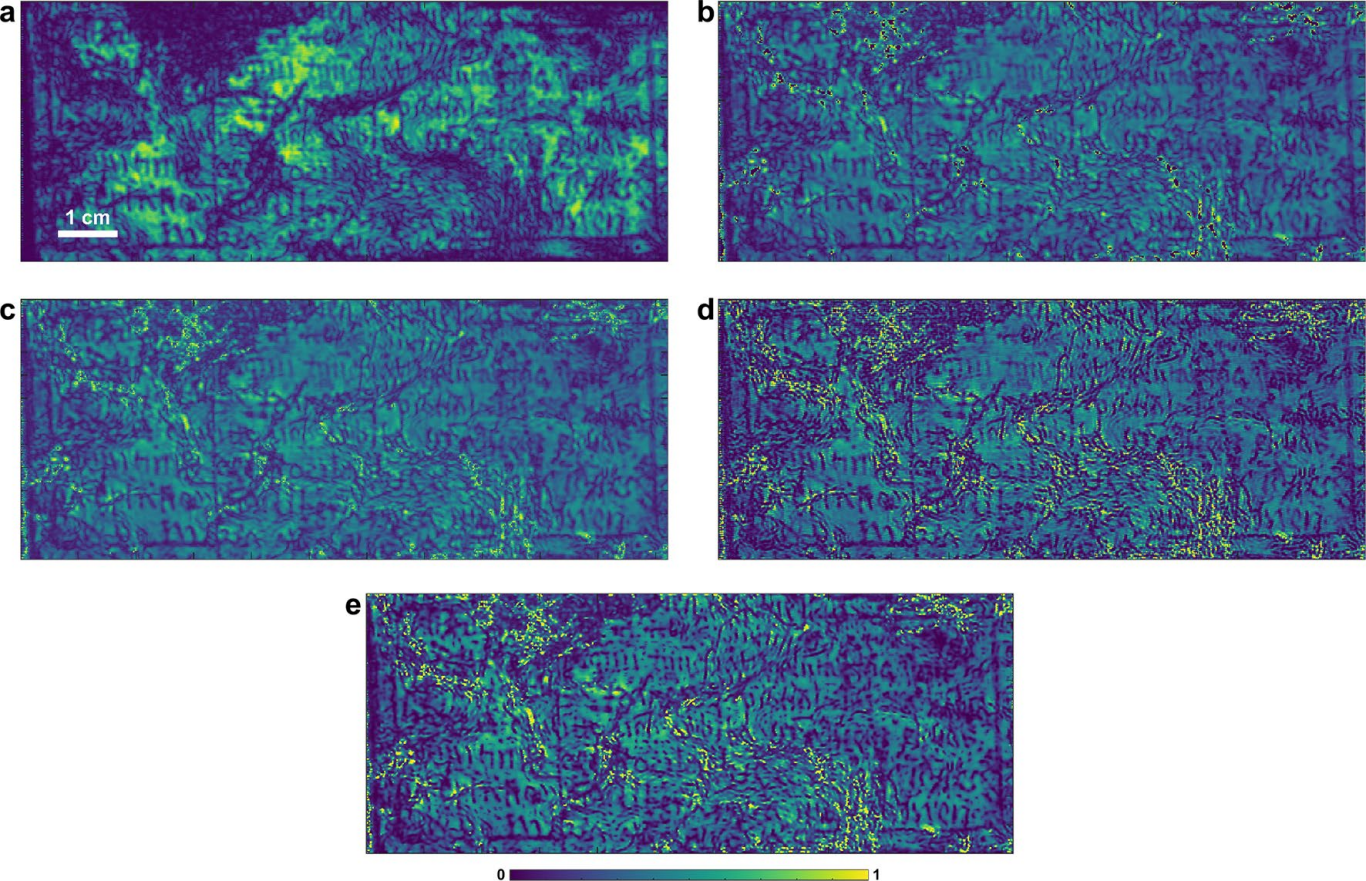
The output images from each step of the post-processing chain. (**a**) The input image $R_{in}(x,y)$. (**b**) The image after intensity correction. (**c**) The image after inpainting. (**d**) The image after blind deconvolution. (**e**) The image after contrast enhancement. All the images are normalized to their maximum pixel values.

Based on the achieved THz multispectral images, we find out that, although observable in the high-frequency bands, the inscriptions suffer from severe degradation due to corrosion; in addition, the image contrast of the inscriptions is not high due to the relatively low spatial resolution provided by THz measurements. Therefore, digital image restoration and enhancement are required to improve the legibility of the inscriptions. In principle, the image of such a corroded Pb cross can be considered as a combination of several superimposed layers of information, including the background layer, the inscription layer, and the degradation layer. The latter is assumed to have been mixed with the background and inscription layers during the corrosion process. In particular, the background and inscription layers can be separated in the THz multispectral images shown in Fig. 3, because the information related to the inscriptions mainly resides in the high-frequency bands (above 1 THz) and thus, is isolated from the low-frequency bands (below 1 THz). In our study, we consider the THz spectral image in the frequency band between 0.5 and 1 THz (Fig. 3b) as the ’background’ layer $R_{bg}(x,y)$, and the one in the frequency band between 2.5 THz and 3.0 THz (Fig. 3f) as the ’inscription’ layer $R_{in}(x,y)$. Here, we propose a post-processing strategy for image restoration and enhancement, which is able to enhance the features associated with the inscriptions in $R_{in}(x,y)$ while removing undesirable artifacts based on the information provided by $R_{bg}(x,y)$. Our image post-processing chain contains four steps:

1. Correction of intensity variations. Variations in surface morphology of the corroded Pb cross have a strong impact on THz reflections. As shown in Fig. 3, such variations in intensity can be seen in all THz multispectral images. Despite the difference in spatial resolutions, the background image $R_{bg}(x,y)$ shown in Fig. 3b can be considered as a good estimate of the intensity variations due to surface morphology, in which the influence of the inscriptions is excluded. Based on the information provided in $R_{bg}(x,y)$, the intensity variations in $R_{in}(x,y)$ shown in Fig. 4a can be corrected using the following model^31^
  2 $$\begin{aligned} R_{in}^{corr}=\mu _{bg}\frac{R_{in}(x,y)}{R_{bg}(x,y)}, \end{aligned}$$
  where $\mu _{bg}$ is the global mean value of the intensity in $R_{bg}(x,y)$. In this model, the factor $\mu _{bg}/{R_{bg}(x,y)}$ aims to mitigate the bright pixel if $\mu _{bg}<{R_{bg}(x,y)}$, and enhance the dark pixel if $\mu _{bg}>{R_{bg}(x,y)}$, in turn leading to a decrease in the intensity variations. The image after intensity correction is shown in Fig. 4b, in which intensity variations due to the surface morphology are successfully removed.
2. Image inpainting. The objective of inpainting is to reconstruct missing regions in an image. As shown in Fig. 4b, the image after intensity correction contains some black areas with an intensity of zero. This is because the SNR in these areas is low, then the division of small values in the correction model leads to the generation of high spike values, which we have to force to zero. Here, we implement the total variation (TV) inpainting algorithm^31^ to fill the missing areas based on the image information available outside. To simplify the notations in the next formulæ, we use $u_0$ and *u* to represent the images before and after inpainting. The TV inpainting algorithm aims at minimizing the function
  3 $$\begin{aligned} E=\lambda (u-u_0)^2 + \alpha \left| \nabla u \right| , \end{aligned}$$
  where $\lambda$ is a binary mask indicating the missing areas to be filled in. In our study, $\lambda$ is generated by detecting the areas in which $R_{in}(x,y)/R_{bg}(x,y)$ is larger than 2. $\alpha$ is the regularization parameter, which controls the tradeoff between these two terms. To minimize the energy in Eq. (3), we solve the Euler-Lagrange differential equation for *E* with a gradient-descent method and a discretization using finite differences. The iterative update formula^31^ can be expressed as
  4 $$\begin{aligned} u_{ij}^{[n+1]}=u_{ij}^{[n]}+\Delta t \left( \alpha \left[ \frac{u_{xx}^{[n]}u_y^{[n]2}-2u_{x}^{[n]}u_{y}^{[n]}u_{xy}^{[n]} +u_{yy}^{[n]}u_{x}^{[n]2}}{\left( u_x^{[n]2}+u_y^{[n]2}\right) ^{3/2}}\right] _{ij} -2\lambda (u_{ij}^{n}-u_{0ij}^{n})\right) , \end{aligned}$$
  where $u_x$ and $u_y$ denote the row and column derivative of *u*. *n* is the number of iteration and the subscripts *i* and *j* indicate the pixel position. $\Delta t$ denotes the time step between temporal samples of *u* (any small constant makes the iteration stable). In our study, we choose $\alpha$=100, $\Delta$=0.01, and *n*=100. The image after the implementation of inpainting is shown in Fig. 4c.
3. Resolution enhancement. The spatial resolution of the achieved image can be further improved by deconvolution. Image deconvolution algorithms aim at removing the blurring present induced by the limited spot size of the THz beam. In general, an imaging system is assumed linear and spatially shift invariant. The relationship between the original image *o*(*x*, *y*) and the output image of the system *u*(*x*, *y*) follows the 2D deconvolution theorem
  5 $$\begin{aligned} u(x,y)=h(x,y) \otimes o(x,y) + n(x,y), \end{aligned}$$
  where $\otimes$ denotes the convolution operator. *h*(*x*, *y*) represents the point spread function (PSF) of the system and *n*(*x*, *y*) denotes the random spatial distribution of noise. When PSF is known, the Richardson-Lucy (RL) algorithm^32^ developed based on Bayes’ theorem can be applied, and its iterative representation is given by
  6 $$\begin{aligned} o_{n+1}(x,y)=\left[ \frac{u(x,y)}{h(x,y)\otimes o_{n}(x,y)}\otimes h(-x,-y) \right] o_{n}(x,y). \end{aligned}$$
  The initial estimation of the object is set as *u*(*x*, *y*) to start the iteration. When PSF is unknown, it refers to a blind deconvolution regime and thus, two similar RL deconvolution iterations^33^ are required. At the *n*-th iteration, the PSF can be estimated from one iterative branch and then is substituted into the other branch to find the estimated image. Such iteration representations are shown as
  7 $$\begin{aligned} h_{n+1}(x,y)=\left[ \frac{u(x,y)}{h_{n}(x,y)\otimes o_{n}(x,y)}\otimes o(-x,-y) \right] h_{n}(x,y), \end{aligned}$$
  8 $$\begin{aligned} o_{n+1}(x,y)=\left[ \frac{u(x,y)}{o(x,y)\otimes h_{n+1}(x,y)}\otimes h_{n+1}(-x,-y) \right] o_{n}(x,y), \end{aligned}$$
  where a uniform illumination can be used as the initial guess of PSF. In our study, we use 100 times of iterations and a deblurred image with higher spatial resolution is achieved, as shown in Fig. 4d.
4. Contrast enhancement. An intuitive way to enhance the contrast of the inscriptions is to set the intensity of the areas considered as inscriptions to zero. Here, we propose to use Niblack’s method^34^ to enhance the contrast, which is a pioneering adaptive thresholding method for document image binarization. This method provides a local threshold value $T_{Niblack}(x,y)$ for each pixel,
  9 $$\begin{aligned} T_{Niblack}(x,y)=\mu (x,y) + k \sigma (x,y), \end{aligned}$$
  where $\mu (x,y)$ and $\sigma (x,y)$ are the mean and standard variation of the intensity on a sampling window around the pixel at (*x*, *y*). Any pixel is labelled as an inscription pixel if $u(x,y)<T_{Niblack}(x,y)$, and is assigned an intensity value of zero. The size of sampling window is critical and actually depends on the average distance between the distinct sites of information, corresponding to the width of the strokes of the inscriptions. In our study, the sampling window is a neighborhood of $3 \times 3$ size around the given pixel at (*x*, *y*). The parameter *k* controls the behavior of the method and a negative value of − 0.8 is used to allow the method to capture the weakened parts of the strokes. The image finally achieved after the post-processing chain is shown in Fig. 4e.

## Discussion

**Figure 5:**
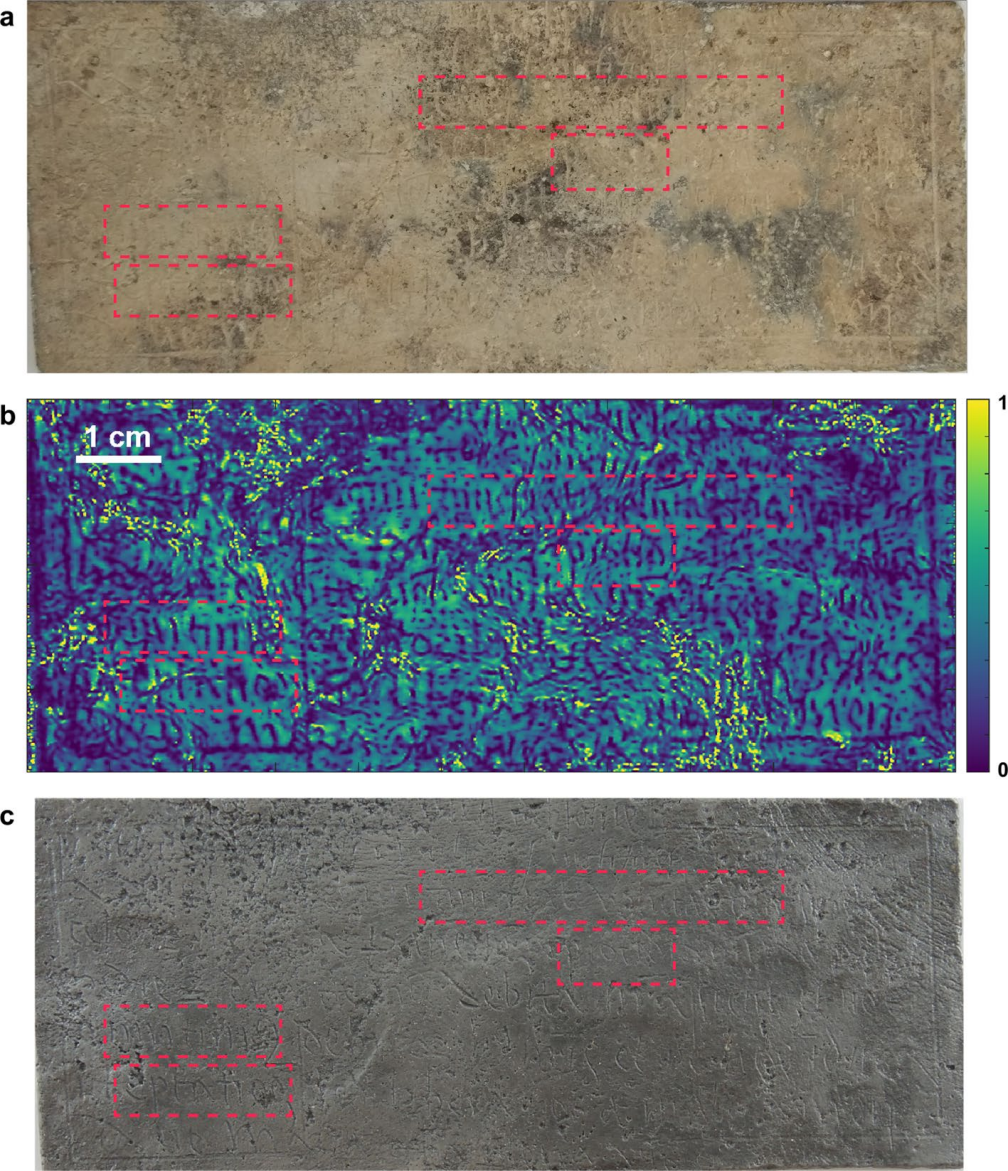
Comparison between the optical and THz images regarding the visualization of inscriptions. (**a**) Photograph of the original Pb cross. (**b**) Final THz image of the Pb cross after post-processing. (**c**) Photograph of the Pb cross after chemically removal of the corrosion. Typical regions are highlighted, in which inscriptions are revealed in the THz image but are barely visible in the optical image before removing the corrosion. As can be read from the THz image (**b**) and confirmed after removal of corrosion (**c**), the inscriptions in the top right boxes are *‘tuum fiat voluntas tua’* and part of *‘quotidianum’*; the ones in the bottom left boxes are a portion of *‘imittimus’* and *‘tentationem’*, respectively.

The final THz image achieved after post-processing indeed reveals a significant portion of features associated with the inscription. To verify our results to the extent possible, the corrosion on the Pb cross was subsequently electrochemically reduced (see “Methods”), and we compare the THz image with the optical images acquired before and after reversed the corrosion, as shown in Fig. 5. It is clear that the THz image (Fig. 5b) is able to uncover almost all the features that can be observed after removing the corrosion (Fig. 5c). In particular, as shown in the highlighted regions in Fig. 5, the THz image successfully reveals some inscriptions that were barely observable from the original Pb cross (before removing the corrosion, as shown in Fig. 5a). Based on the observed features, we can tell that the inscriptions were engraved in cursive Carolingian minuscule and the language is Latin. Still, reading the inscription requires specialists. Specifically, our co-author from the Musée Charles de Bruyères has identified the Latin words *‘tuum fiat voluntas tua’* and part of *‘quotidianum’*, based on the THz features in the top right red rectangular boxes, and a portion of *‘dimittimus’* and *‘tentationem’*, based on the THz features in the bottom left boxes, respectively. All these words come from the *Pater Noster* (Lord’s Prayer) and their reading is confirmed by the examination of the cross after the corrosion products have been removed. *Pater Noster* is not obviously an absolution prayer, as noted in Ref.^2^, none of the crosses in the Remiremont collection contain obvious absolution formulæ. Consequently, specialists also refer to these plaques as *des croix d’identités* as many of their inscriptions refer to the deceased. Our results demonstrate the effectiveness of THz imaging for the visualization of obscured inscriptions on a corroded Pb cross in a nondestructive and noncontact manner. It is noteworthy that the success of revealing obscured inscriptions relies on the extraction of the high-frequency components of a broadband THz pulse. At the same time, even though the low-frequency bands lack the resolution to fully capture the detail of the inscription itself, these bands were needed to remove confounding extraneous features from the image. Images based on time-domain information, such as peak values of the reflected signals, appear to be entirely useless for the perspective of our aim to uncover the inscriptions.

In summary, we have explored the potential of THz multispectral imaging as an archæometric approach to reveal the inscriptions on a sixteenth century Pb funerary cross from Remiremont. We have demonstrated that, by taking advantage of the broadband nature of a THz pulse, we are able to characterize the Pb cross at various spatial resolutions and visualize the inscriptions in a nondestructive and noncontact fashion under an obscuring corrosion layer. In particular, our results have also shown that the legibility of the inscriptions achieved by physical measurements can be further improved by digital image restoration and enhancement. Our THz images reveal major aspects of the inscriptions are hitherto undocumented and thus will contribute not only to the understanding of this cross but also to the sixteenth century Lorraine ecclesiastic epigraphy. As a personal remark, our contribution required a truly multidisciplinary effort, with the collaborations of museums, conservation laboratories, medieval historians, and engineers. The cross studied is one of a large collection of such objects from the Musée Charles de Bruyères in Remiremont. Other large collections are held elsewhere, notably in the Tresor de la Cathedrale Saint-Étienne in Metz, France. Many objects in these collections are still in their corroded state. It is worth noting that, Pb was widely used for archæological objects, such as sarcophagi and for plaques on monuments, not to mention plumbing, where it may sometimes not be feasible to reduce corrosion layers on some of these artifacts. Therefore, the proposed THz imaging modality can be considered as an effective alternative for the interpretation of inscriptions and details on the surface of Pb objects obscured by a corrosion layer. Our approach can also be applied for a broad range of archæological objects and is envisioned to provide unprecedented information for art-historical studies, as well as documentation, restoration, and conservation.

## Methods

### THz imaging system

A typical THz time-domain system (Teraview TPS Spectra 3000) operating in a reflection geometry was employed in this study. The incident angle of the THz beam was $\sim$10$^\circ$C. The GaAs photoconductive antenna was excited by an ultrafast laser to produce roughly single-cycle THz pulses with bandwidth extending from 60 GHz to 3 THz. The maximum peak of its power spectrum was located at about 0.3 THz. Each recorded temporal reflected THz waveform contains 4096 data points, and the data sampling period was set to 0.011634 ps. The signal was averaged over 10 shots per pixel to enhance signal to noise. The scanning of the sample was conducted in a temperature-controlled laboratory at 22 $^\circ$C. The humidity in the laboratory was held about 38$\%$.

### FWDD algorithm

FWDD algorithm^35^ was employed in order to accurately measure the optical delay between the echoes from the air/corrosion and corrosion/Pb interfaces. In the time domain, the reflected THz signal *r*(*t*) is the convolution of the incident THz pulse *i*(*t*) with the impulse-response function *h*(*t*), which corresponds to the structure and properties of the sample at a given position. Ideally, *h*(*t*) can be retrieved by the inverse Fourier transform based on the convolution theorem. However, successful deconvolution cannot be expected by directly applying the inverse Fourier transform, since division by small numbers in the frequency domain will give rise to large spikes in the high frequency region^36^, in turn leading to severe ringing in the time domain. Therefore, frequency-domain filtering is introduced to suppress the high-frequency noise. Then *h*(*t*) can be expressed as:

10 $$\begin{aligned} h(t)=FFT^{-1}\left[ FFT(f(t))\times \frac{FFT(r(t))}{FFT(i(t))}\right] \ , \end{aligned}$$

where *f*(*t*) corresponds to the filter function in the time domain. In this study, a von Hann window function is chosen as the filter function *f*(*t*), and its frequency spectrum $F(\omega )$ can be expressed as,

11 $$\begin{aligned} F(\omega )= {\left\{ \begin{array}{ll} e^{i\omega t_0}cos^2(\frac{\omega }{4f_c}), &{} \left| \omega \right| <2\pi f_c \\ 0, &{}\left| \omega \right| \ge 2\pi f_c \end{array}\right. } \ , \end{aligned}$$

where $t_0$ corresponds to the arrival time of the THz main peak in time and $f_c$ is the cut-off frequency. The selection of $f_c$ is a compromise between the time resolution and frequency-domain filtering. Usually, a relatively high value of $f_c$ is selected ($f_c$ = 4 THz in this study) in order to achieve a high resolution in time. However, in this case, a satisfactory signal-to-noise ratio in time cannot be guaranteed. Therefore, stationary wavelet shrinkage is applied to further attenuate the residual noise in time. The *symlet* (sym4) wavelets are chosen in this study. A maximum level of 7 is used for the wavelet decomposition as no significant improvement can be observed for higher levels to justify the extra computational expense^37^. Quite often, the signal after FWDD contains slow fluctuations corresponding to the low-frequency noise, due to the THz source being inefficient in the low frequency region. This kind of noise can be cancelled by subtracting the baseline of the deconvolved signal.

### Electrolytic reduction of lead corrosion products

The electrolytic treatment^3,38^ applied to the lead cross consists in polarizing the lead structure as a cathode to obtain the reduction of corrosion products. The electrolyte used is neutral and composed of 2$\%$ sodium sulfate in tap water. The electric potential of the cross has been monitored throughout the treatment. At the end of the treatment, the surface appears metallic, slightly grayish, the reliefs and decorations are preserved and more easily visible. Of note, in addition to cleaning, consolidation is obtained thanks to the reduction of the lead oxides which crack the structure.
